# Non-spike protein inhibition of SARS-CoV-2 by natural products through the key mediator protein ORF8 

**DOI:** 10.22099/mbrc.2024.50245.2001

**Published:** 2025

**Authors:** Mostafa Bagheri-Far, Mohammad Assadizadeh, Maryam Azimzadeh-Irani, Mohammad Yaghoubi-Avini, Seyed Massoud Hosseini

**Affiliations:** Faculty of Life Sciences and Biotechnology, Shahid Beheshti University, Tehran, Iran

**Keywords:** SARS-CoV-2, non-spike protein, ORF8, Molecular Docking, Natural Products

## Abstract

The recent pernicious COVID-19 pandemic is caused by SARS-CoV-2. While most therapeutic strategies have focused on the viral spike protein, Open Reading Frame 8 (ORF8) plays a critical role in causing the severity of the disease. Nonetheless, there still needs to be more information on the ORF8 binding epitopes and their appropriate safe inhibitors. Herein, the protein binding sites were detected through comprehensive structural analyses. The validation of the binding sites was investigated through protein conservation analysis and blind docking. The potential natural product (NP) inhibitors were selected based on a structure-function approach. The solo and combined inhibition functions of these NPs were examined through molecular docking studies. Two binding epitopes were identified, one between the ORF8 monomers (DGBM) and the other on the surface (Gal1-Like). E92 was predicted to be pivotal for DGBM, and R101 for Gal1-like, which was then confirmed through molecular dockings. The inhibitory effects of selected phytochemical (Artemisinin), bacterial (Ivermectin), and native-liken (DEG-168) NPs were compared with the Remdesivir. Selected NPs showed solo- and co-functionality against Remdesivir to inhibit functional regions of the ORF8 structure. The DGBM is highly engaged in capturing the NPs. Additionally, the co-functionality study of NPs showed that the Ivermectin-DEG168 combination has the strongest mechanism for inhibiting all the predicted binding sites. Ivermectin can interfere with ORF8-MHC-I interaction through inhibition of A51 and F120. Two new binding sites on this non-infusion protein structure were introduced using a combination of approaches. Additionally, three safe and effective were found to inhibit these binding sites.

## INTRODUCTION

The coronavirus disease 2019 pandemic, caused by the Severe Acute Respiratory Syndrome Coronavirus 2 (SARS-CoV-2), emerged as an imperative challenge and revealed the need for revolutionary therapeutic approaches [1, 2]. Resembling several other β-coronaviruses such as SARS-CoV-1 and MERS-CoV, the potential of the SARS-CoV-2 virus to cause diversified disease signs in humans is noteworthy [3]. Open Reading Frame 8 is among the SARS-Cov-2 accessory proteins. ORF8 protein is an interesting and promising candidate to be considered in the quest for antiviral drugs [4]. 

SARS-CoV-2 ORF8 mutations such as Δ382, L84S, V62L, and D119 and F120 deletions cause less severe inflammation and an attenuated disease phenotype of COVID-19 in consequences [5-11]. ORF8 emerges as a pivotal player in the complex interplay between SARS-CoV-2 and human host cells [12, 13]. Studies have unveiled a plethora of functions that ORF8 undertakes within the host, encompassing disruptions to the host cell machinery, immune system regulation and evasion, as well as direct harm inflicted on various host organs. Specifically, it triggers Endoplasmic Reticulum stress (ER-stress) through various mechanisms and perturbs chromatin regulation [9, 14-19]. Additionally, ORF8 exerts a substantial impact on immune system regulators and adeptly facilitates immune evasion by directly curtailing the exportation of Major Histocompatibility Complex-1 (MHC-I) to the cell surface [19, 20]. Furthermore, it wields control over cytokine expression, with some instances leading to severe consequences, including fatality [16, 21-24]. 

Its significance reverberates across various aspects of the host-virus interaction landscape, making it a vital focal point for further investigation. Accordingly, SARS-CoV-2 ORF8 is a suitable target for novel therapeutics and vaccination strategies evolution that is currently focused on spike protein [12, 13, 25].

It has been observed that individuals infected with SARS-CoV-2 have a high level of ORF8 in their blood. Additionally, the secretory nature of ORF8 has been proven [21, 26]. Wenzhong and Hualan have predicted that ORF8 may use the hemoglobin Heme group for nitric oxide synthesis [27]. This could lead to the removal of the oxygen carrier from the respiratory gas exchange system [28]. There is also evidence to suggest that COVID-19 patients infected with mutated ORF8 variants experience reduced hypoxia [7, 29].

**Figure 1:**
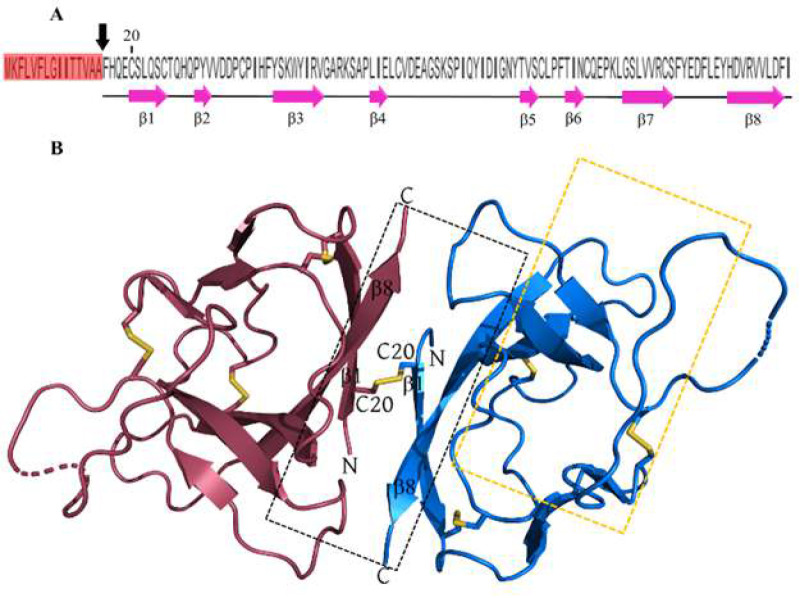
ORF8 sequence and its Ig-like fold homodimeric structure. A) ORF8 121 aa sequence. The signal peptide has been highlighted with salmon and the cleavage site is indicated by a black arrow. Secondary structure assignments (pink cartoon arrows) represent the β-sheets of ORF8 monomer formed by β1 to β8. B) Structure of the ORF8 homodimer is shown with cartoon representation (PDB ID: 7JTL). ORF8 monomer chains are colored as marine and raspberry for chains A and B, respectively. Intra and intermolecular disulfides have been shown in the stick. The covalent interface that is mediated by intermolecular C20-C20 disulfide has been indicated within the dashed black box. The non-covalent interface scope has been indicated within the dashed golden box. The N and C-terminal have been marked by N and C letters, respectively.

SARS-CoV-2 ORF8 presents a structural profile as an Immunoglobulin-like homodimeric protein. Its monomers are linked through a Cys20-20Cys disulfide bond and each of them is composed of 121 amino acids. Notably, there is a cleavable signal peptide (SP) at the N-terminal, as illustrated in Figure 1A [30, 31]. Furthermore, the Ig-like fold of the protein, as seen in Figure 1B, has a distinctive region within the loop between beta 4 and beta 5 strands. this loop contains a 73YIDI76 motif at the center. This unique region can form a non-covalent interface, enabling ORF8 to construct high-order assemblies (Fig. 1C) [31, 32]. These structural attributes distinguish SARS-CoV-2 ORF8 from its relatives and contribute significantly to the functions exhibited by this non-infusion protein.

Remdesivir is the only approved antiviral drug to treat COVID-19 that targets SARS-CoV-2 RNA-depended RNA polymerase (RdRp) protein [33-36]. Considering recent challenges to the clinical efficacy of Remdesivir, it is important to explore alternative treatment options for COVID-19 patients, particularly with non-toxicity and low risk. Natural products (NP) compounds fitting these criteria emerge as valuable, low-risk assets against the virus [37-40]. Furthermore, the diverse structural landscape of NPs lends itself well to computational approaches, which can expedite the screening of potent NP inhibitors. These innovative methods introduce potential therapies that hold promise for controlling the pathogenicity of SARS-CoV-2 [37-40].

In this study, in-silico predictions, integrating structural visualization, electrostatic mapping, and ASA calculations were utilized to identify key binding sites and epitopes within ORF8. Structural approach, verified through sequence alignment and extensive analysis, pinpoints a complex binding site between the monomers as well as other binding sites highlighted by the I-TASSER server [41]. The validity of predictions was confirmed by a conservation study through sequence alignment of 22947 SARS-CoV-2 ORF8 sequences. Three promising potential inhibitors, Artemisinin [42-49], Ivermectin [50-58], and DEG-168 [59, 60] have been selected based on their safety profiles, ORF8 structure-function features, and I-TASSER server predictions. These inhibitors were assessed and compared with Remdesivir against ORF8 dimer in molecular docking, revealing their efficacy in inhibiting the key binding sites of ORF8.

## MATERIALS AND METHODS

**Data Sets Retrieval:** *SARS-CoV-2 ORF8 Reference Sequence and its Structure:* The SARS-CoV-2 ORF8 reference sequence (YP_009724396.1) was gathered from GenBank [61]. The three-dimensional (3D) structure of SARS-CoV-2 ORF8 (PDB ID: 7JTL) was obtained from the Protein Data Bank (PDB) [62]. 

**ORF8 Sequences of circulating variants of SARS-CoV-2:** In addition to the ORF8 reference sequence, 22946 sequences of all circulating variants of SARS-CoV-2 ORF8 were obtained from NCBI in order to validate the prediction of binding sites by sequence alignment and conservation study. The time interval was from January 13, 2020, to January 28, 2022. The filters used included setting ambiguous characters to zero and nucleotide completeness to complete. 

**Target Preparation for Structural Analyses and Molecular Docking Studies:** The homodimeric X-ray structure of 7JTL was prepared for docking studies. The missing residues including _65_AG_66_ in chain A and _66_GSK_68_ in chain B, were modeled in PyMOL^©^, version 2.5.2. Then, these modeled regions were computationally refined in the ModLoop server [63]. Subsequently, this structure was minimized through UCSF Chimera 1.15 software [64]. Minimization was carried out in two successive stages for all atoms. Initially, the minimization was performed using the steepest descent method for 2000 steps, followed by the conjugate gradient minimization for 5000 steps [65]. 

**Ligands Structures Preparation:** The 2D and 3D structures of the Remdesivir (CID: 121304016) and the phytochemical Artemisinin (CID: 68827) were retrieved from the PubChem server in Simple Data File (SDF) format [66]. The 2D structure of the Microbial Metabolite Ivermectin was sourced from PubChem (CID: 6321424), while its 3D structure was acquired from the Protein Data Bank (PDB ID: 5YDI). For the NP-inspired Lactoside DEG-168, the ChemBioOffice 14.0 software was employed to generate the 2D structure and obtain the 3D structure [67]. The obtained structures of the ligands were converted to MOL2 format by UCSF Chimera 1.15 software for use in molecular docking studies.

**Prediction of SARS-CoV-2 ORF8 Potential Binding Sites:** ***Structural Analysis:*** The full-length minimized ORF8 was utilized in computing both the electrostatic map and the Accessible Surface Area (ASA). The PyMOL Adaptive Poisson-Boltzmann Solver (APBS) plugin was employed for the calculation of the electrostatic map [68]. Parameters were set with an electrostatic potential range of -/+3, while all other settings remained at their default values. The purpose of generating electrostatic maps is to reveal the distribution of electric potential within a solution, which can significantly affect molecular interactions. The Accessible Surface Area (ASA) of the protein was measured online in the Accessible Surface Area and Accessibility Calculation for Protein (ver. 1.2) (http://cib.cf.ocha.ac.jp/bitool/ASA/). The ASA highlighted key residues in the binding sites and binding pockets.  

**Online in-silico Prediction:** The Iterative Threading ASSEmbly Refinement (I-TASSER) server was used for protein binding capacity prediction [41,69,70]. For this purpose, a complete ORF8 reference sequence (YP_009724396.1) was submitted to this server. A combination of methods including structure comparison and protein-protein networks in this server were utilized to function annotations of protein binding sites. Then, the binding site residues were selected based on the templates with the highest C-score. C-score is the confidence score of prediction and it ranges from 0 to 1, where a higher score indicates a more reliable prediction. Furthermore, the potential ORF8 native ligands were detected based on these predictions.

**Validation of Predicted Binding Sites:** To validate the predicted binding sites, a two-step approach was followed. Initially, the sequence alignment of 22947 ORF8 sequences was performed using the MUSCLE algorithm on MEGA7 software [71]. Afterward, the percentage of consensus sequences and the level of conservation of key residues were investigated by the AMSA algorithm, using Jalview software [72, 73].

**Natural Products Screening: **The selection of potential NP inhibitors was based on several factors. These included the surface topology and structure of ORF8, the ORF8 binding sites that interact with host proteins, and the function of secreted ORF8 in the blood. Additionally, approved or safe NPs were considered, along with the predictions of the I-TASSER server.

The involvement of these factors in the potential NP inhibitors selection was in the form of the following three approaches which were used to avoid the need for screening a large number of ligands and save time: 

1. A safe NP whose mechanism of action is in blood and related to Heme could possibly be a potential inhibitor to interfere with the functions of SARS-CoV-2 ORF8 [28, 74-77].

2. The distinct flexible 46-83 region and covalent interface of ORF8 proposed to involve in a large number of protein-protein interactions (PPI) with host cells underscore them as potential targets for drugs [31, 32]. Macrocyclic compounds, which often do not adhere strictly to the criteria set by Lipinski's rule of 5, possess a spherical or disc-like structure that allows them to bind well to this type of protein targets and effectively control them [78-81]. Therefore, a safe NP of this class of compounds is probably a suitable ligand to control ORF8.

3. Based on the functional annotation and suggested protein templates of I-TASSER server (Table 1 and supplementary, Table S3), NP inhibitors of these templates were considered as potential inhibitors for ORF8. 

Lastly, Three well-known NPs, the plant substance Sesquiterpene lactone Artemisinin (CID: 68827) (using approaches 1 and 3) [45,82,83], the microbial macrocyclic metabolite Ivermectin (CID: 6321424) (using from approach 2) [84, 85], and the natural ORF8 ligand-like substance, lactoside DEG-168 (using approach 3) was investigated as selective possible inhibitors of ORF8 [86]. Additionally, the inhibitory competition of Remdesivir (CID: 121304016) [87-89], the only approved anti-SARS-CoV-2 drug, was examined with these three selected NPs.

**Molecular Docking study:** Molecular docking of NP molecules to ORF8 was conducted by Autodock Vina 1.1.2 in UCSF Chimera software 1.15 [64, 90, 91]. Binding poses with the highest affinity scores for each NP were investigated. Two distinct methods, namely semi-flexible docking and blind docking, were utilized. Blind docking allows the entire surface of the protein to be searched by ligands leading to a structural validation of binding sites [92]. Semi-flexible docking also allows the rigid parts of the ligand to move around the rotatable bonds and adopt all possible conformations for binding to the receptor. The interaction of ORF8-NP complexes was analyzed through the Protein-Ligand Interaction Profiler’s (PLIP) webserver [93, 94]. The minimum cut-off of 4.1 Å was set for H.bonds to show the maximum inhibitory capacity of small molecules. 

**Visualizations: **All molecular visualizations were created using PyMOL version 2.5.2, and statistical calculations and graphs were generated using Excel 2016 [95, 96].

## RESULTS

**Figure 2:**
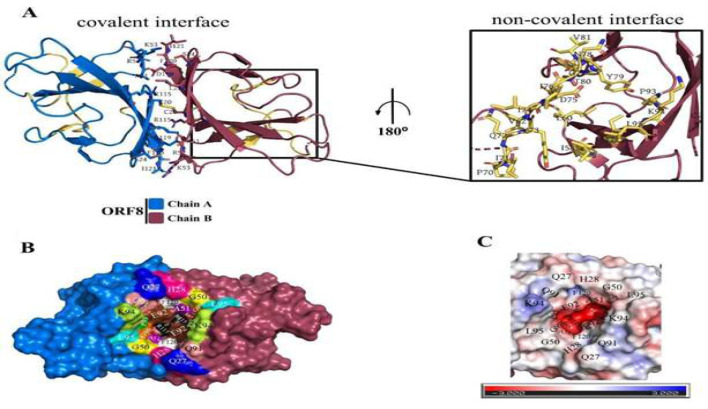
Covalent and non-covalent interfaces and their relationships. A) The engaged residues of covalent and non-covalent interfaces of SARS-CoV-2 ORF8 have been displayed on sticks and labeled for PDB ID 7JTL. The golden area on each chain indicates the unique region of aa 46-83. B) The surface diagram of the residues that formed the Deep Groove Between ORF8 Monomers (DGBM) has been presented on the modeled and minimized ORF8 structure PDB 7JTL, which was prepared earlier for docking studies. The accessible surface of each residue is shown with different colors and labeled. The SARS-CoV-2 ORF8 chains have been colored marine and raspberry for chains A and B, respectively. C) Electrostatic mapping of DGBM. The approximate scopes of DGBM-forming residues have been indicated by the name of residues. The charging guide has been shown below the map so that the red color indicates the negative charge, white is neutral, and blue is the positive charge.

The SARS-CoV-2 ORF8 possesses two oligomerization interfaces consisting of specific amino acid residues [31]. A deep groove between the monomers (DGBM) is assembled by some of these specific residues including A51, R115, and F120, from the covalent interface, and 93PKL95 from the non-covalent interface, in addition to Q27, H28, G50, Q91, E92, G96, and S97 (Fig. 2 B).

Calculating the electrostatic map of the DGBM revealed the highly polar nature of this pit, with a noticeable negative charge in the lower part due to the presence of G50, A51, E92, P93, G96, S97, F120, and especially R115, and a low positive polarity in the upper part originating from H28, Q91, and K94 (Fig. 2 C).

Notably, E92 residues of both monomers are particularly significant, as they are entirely situated within the DGBM, have a considerable accessible surface area (∼ 89 Å2), and exhibit polarity (Fig. 2 C).

Given these findings, the DGBM appears to play a crucial role in capturing ligands that offer the opportunity to destabilize the covalent interface. In line with previous studies suggesting that ORF8 dimer instability can lead to disruption of ORF8 direct interaction with MHC-1, targeting DGBM could interfere with the interaction network of accessible residues and eliminate functions dependent on the interface's native conformation [32,97].

7JTL. The golden area on each chain indicates the unique region of aa 46-83. B) The surface diagram of the residues that formed the Deep Groove Between ORF8 Monomers (DGBM) has been presented on the modeled and minimized ORF8 structure PDB 7JTL, which was prepared earlier for docking studies. The accessible surface of each residue is shown with different colors and labeled. The SARS-CoV-2 ORF8 chains have been colored marine and raspberry for chains A and B, respectively. C) Electrostatic mapping of DGBM. The approximate scopes of DGBM-forming residues have been indicated by the name of residues. The charging guide has been shown below the map so that the red color indicates the negative charge, white is neutral, and blue is the positive charge.

The I-TASSER server is also utilized to predict the potential binding sites of SARS-CoV-2 ORF8. The two templates with the highest Coefficient scores (C-score) include human Galectin-1 (Gal-1) and Galbeta1-4(6OSO3)GlcNAc complex and Catalytic elimination antibody 34E4 in complex with hapten (Supplementary, Table S2).

**Figure 3:**
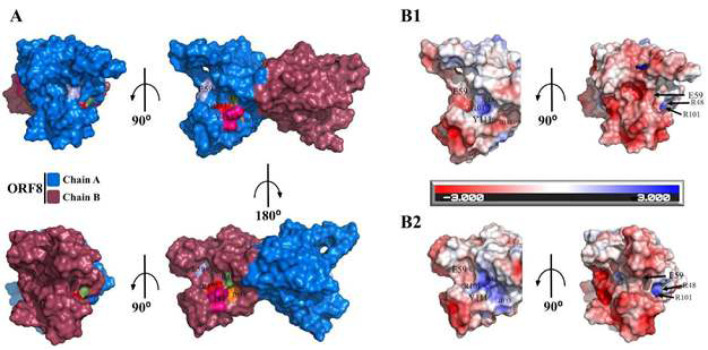
Structural analysis of I-TASSER predicted binding site residues. A) The region colored with different color codes and labeled on the ORF8 dimeric structure surface indicates hotspot residues predicted by the I-TASSER server. The dimeric structure of SARS-CoV-2 ORF8 is shown in the surface diagram, and its chains are colored navy and raspberry for A and B chains, respectively. B1, 2) APBS electrostatic calculation. An electrostatic map for the Gal1-like binding site and its residues is shown. The guide of charging is shown between figures B1 and B2, where red, blue, and white colors represent negative, positive, and neutral charges, respectively, in the range of -/+3.

Based on these templates, six interacting residues of ORF8 are identified as potential binding residues including R48, E59, R101, Y111, D113, and R115. These residues except R115 form an interface hereafter called the Gal1-like binding site. R115 is excluded because it is solely accessible from the DGBM region (Figs. 1-3b and 3-2). Consequently, it is considered part of the DGBM binding site. Both R48 and E59 are located in the flexible region of ORF8 (residues 46-83) centered by the noncovalent oligomerization interface [31] (Fig. 2 A). R48, R101, Y111, and D113 are located on the surface of the ORF8 chains and the electrostatic map reveals that these residues are responsible for the positive polarity of this region. On the other hand, E59 causes a negative polarity on chains (Fig. 3 B1 and B2).

Ligand-binding sites may be conserved since conserved residues are more likely to be functionally important. Accordingly, the conservation of 22947 SARS-CoV-2 ORF8 sequences was examined to support the binding site prediction study (109, 110). The AMSA conservation scores for the predicted binding residues were 10 and 11 (out of 11), except for F120 with a score of 2. Additionally, The consensus scores for predicted binding residues were above 97%, except for F120 with a score of 85%. These results revealed that the predicted DGBM and Gal-like binding sites are highly valid (Table 1). 

**Table 1:** Predicted hotspots and their conservation.

**No**	**BINDING SITE**		**BINDING SITE RESIDUES**	**MUTATION** **S**		**CONSERVATION (0-11)**	**∼** **CONSENSUS%**
	**Gal-1 like**	**DGBM**		**SUBSTITUTION WITH**	**FREQUENCY%**		
**1**		●	Q27	**R**	0.004	**10**	**99**
				**K**	0.004		
				**-**	0.992		
**2**		●	H28	L	0.004	10	99
				Y	0.004		
				-	0.992		
**3**		●	G50	-	0.992	11	99
**4**		●	A51	S	1.32	10	98
				V	0.109		
				F	0.013		
				T	0.013		
				-	0.445		
**5**		●	Q91	K	0.009	10	99
				I	0.004		
				L	0.004		
				P	0.004		
				R	0.004		
				-	0.0975		
**6**		●	E92	K	1.286	10	98
				S	0.004		
				-	0.71		
**7**		●	P93	S	2.244	10	97
				L	0.01		
				-	0.746		
**8**		●	K94	E	0.009	10	99
				R	0.004		
				-	0.987		
**9**		●	L95	F	0.253	10	99
				M	0.004		
				S	0.004		
				V	0.004		
				-	0.735		
**10**		●	G96	D	0.004	10	99
				-	0.996		
**11**		●	S97	I	0.009	2	85
				G	0.004		
				-	0.987		
**12**		●	F120	L	0.689	10	99
				K	0.022		
				V	0.017		
				I	0.004		
				-	14.2		
**13**	**●**	●	R115	C	0.065	10	99
				L	0.026		
				P	0.017		
				H	0.013		
				-	0.879		
**14**	●		R48	I	0.004	10	99
				-	0.996		
**15**	●		E59	V	0.013	10	99
				G	0.004		
				K	0.004		
				-	0.979		
**16**	●		R101	L	0.022	10	99
				-	0.978		
**17**	●		Y111	C	0.004	10	99
				-	0.996		
**18**	●		D113	Y	0.004	10	99
				-	0.996		

Conservation has been calculated for 22947 aligned SARS-CoV-2 ORF8 sequences. AMSA scores the level of conservation of amino acid residues from 0-11 (from low to high). A score of 11 indicates grouping with default amino acid property, and a score of 10 indicates mutations that all default amino acid properties are conserved. Frequencies of substitution and missense (represented by "-") mutations were presented for each hotspot amino acid of binding sites Gal-1 and DGBM. These sites were predicted by I-TASSER and other structural analyses, respectively.

By prioritizing safe and validated drugs alongside the consideration of structure-function features of SARS-CoV-2 ORF8 including the predictions from the I-TASSER server, this study rapidly identified three potential NPs. Three groups of compounds were realized as potential ORF8 inhibitors. 

Primarily, the herbal substance Artemisinin (Supplementary, Fig. S1B) is from the group of exceptional Sesquiterpene lactone compounds and contains an unusual peroxide bridge.

Next, the microbial metabolite of Ivermectin (Supplementary, Fig. S1C) is from the group of macrocyclic compounds. It is the oldest approved drug on the World Health Organization's list of essential drugs and is used as an antiparasitic agent. 

Based on the template Galectin-1 (Gal-1) of I-TASSER prediction, ORF8 potential native ligands were selected. Galectin-1 is a β-galactoside-binding protein family member, and it plays a critical role in Human Immunodeficiency Virus type 1 (HIV-1) infection in the human host [59,60]. This role is based on Gal-1 binding to β-galactosides and its homodimeric structure. Thus, β-galactoside compounds could be potential native ligands of ORF8 protein as well. In particular, this hypothesis is further strengthened due to the secretion of ORF8 to the extracellular and humoral fluids and the prediction by Wenzhong and Hualan of the interaction of ORF8 with the SARS-CoV-2 envelop protein [27, 98]. Because while they have investigated the interaction of ORF8 with the non-glycosylated form of the virus envelope protein, this protein has glycosylation sites that have not been well studied and perhaps similar to Gal-1, these interactions with SARS-CoV-2 occurs through sugars on the surface of virus proteins.

Finally, DEG-168 is a putative compound similar to the predicted potential native ORF8 ligand based on the Galectin-1 (Gal-1) template (Supplementary, Fig. S1D). The chemical name of this substance inspired by the NPs is β-D-lactosyl naphthyl sulfone and belongs to the lactoside compounds group. Lactoside DEG-168 is one of the members of a new generation of anti-Gal-1 compounds that safe and effectively inhibit the infection process of the HIV-1 virus through this protein [59, 60]. 

Here, only anti-SARS-CoV-2 FDA-approved drug Remdesivir (Supplementary, Fig. S1A), an NP-inspired compound, was also selected and compared for its inhibitory competition mechanism with the above well-known NPs.

**Figure 4:**
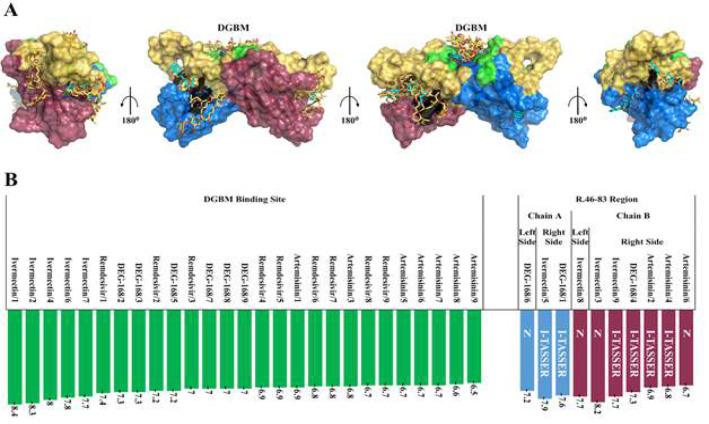
Overall view of NPs inhibitory performances for predicted hotspots. A) All 36 binding poses of four proposed NPs are presented on the dimeric structure of ORF8. Remdesivir, Artemisinin, Ivermectin, and DEG-168 are shown in very peri, smudge, gold, and cyan sticks, respectively. The region highlighted in gold on the protein chains represents the unique region R.46-83. The region highlighted in green and black on the protein indicates the DGBM and Gal1-like binding sites. B) The binding poses have been ordered according to their localization onto ORF8 structure (unique DGBM/R.46-83 regions) and binding affinities (from maximum to minimum in Kcal/mol). In the R.46-83 category, marine and raspberry columns indicated chains A and B, respectively. The binding poses that are localized onto the right/left side of R.46-83 have been marked with R for right and L for left. The letter N on the columns refers to the binding poses in which the predicted residues are not inhibiting. The dimeric structure of ORF8 is presented in marine and raspberry cartoons (for chains A and B, respectively) and transparent surfaces.

Analyses at the Level of ORF8-NPs Interactions Also Led To Confirm The Validity Of The Prediction Of Binding Residues And Effectiveness Of Suggested NPs:

Structures of all the complexes obtained from the molecular docking study were examined (Fig. 4 A). The classification of the poses based on binding region and affinity scores, shows the two dominant regions. The NPs docked on the DGBM polar pit and Gal1-like binding site in 88.9% of complexes. These regions were also identified as potential binding sites in the I-TASSER and surface studies. Therefore, the results of the blind docking study were quite in line with the I-TASSER and other structural predictions. 

It is important to note that the most frequent binding modes for all four ligands (72.2% of complexes) are predicted on DGBM (Fig. 4). However, DGBM also shows significance compared to the rest of the binding sites. The Gal1-like was the most efficient inhibitor of DEG-168. On the other hand, the complexes with the highest affinity score for the other NPs were constructed on the DGBM. Artemisinin, Ivermectin, and DEG-168 exhibit greater flexibility and structural capacity than Remdesivir for binding to various parts of the ORF8 structure, particularly inhibiting the Gal1-like binding site.

Ivermectin exhibited higher potency in inhibiting ORF8 compared to the other three NPs, attributed to diverse binding modes and more negative binding free energies (-7.7 to -8.4 kcal/mol). 

**Figure 5:**
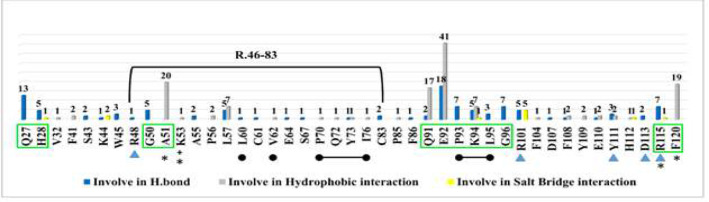
All involved residues in interactions with the four proposed NPs and their repetition rates are shown on a column chart. The repetition rate of each residue in interactions is written on top of columns. The predicted binding site DGBM residues are distinguished inside the green boxes, and predicted residues by the I-TASSER server (Gal1-like binding site) are indicated with ∎. Residues distinguished with ● or * are important in the non-covalent and covalent interfaces, respectively. The horizontal bracket determines the ORF8 unique region of residues 46-83.

A general statistical analysis of the participation rate of ORF8 interacting residues with NPs is presented in Figure 5. Interacting residues are specified based on PLIP analysis profiles at the atomic level for the complexes resulting from NPs docking with ORF8.

The statistical analysis shows that all residues of the two predicted binding sites (Table 1) (except for S97 of the DGBM binding site and E59 of the Gal1-like binding site) were among the 45 interacting residues and collectively contributed to 78% of the interactions (Figure 5). These statistics directly indicate the significant importance of these two binding sites for ORF8 functions and thus their suitability as drug targets in the ORF8 structure. Figure 5 illustrates that the binding residue E92 of the predicted DGBM binding site is highly involved in numerous hydrophobic and hydrogen interactions. It has a significant number of interactions, particularly in hydrogen bonds, which play a crucial role in the stability of the formed complexes. Additionally, it is demonstrated that binding residue R101 is more involved in Gal1-like binding site interactions.

Molecular docking provided mechanistic details of ORF8–NP interactions, which assisted in a more accurate exploration of their inhibiting pattern (Supplementary, Fig. S2) that further supported identifying the key interacting residues (Table S3). 

The solo-functionality pattern of the suggested NPs was explored by investigating the optimal binding modes for each NP. The optimal binding modes were considered to be those in which the predicted binding sites were inhibited with the highest level of predicted affinity (Supplementary, Fig. S2).

Based on the interaction profile of ORF8-NP complexes, Remdesivir (Figures 8 and Tables S2 to S5) has a stronger solo functionality for DGBM inhibition compared to other natural products. Remdesivir shows specificity for DGBM by binding only to this site, despite other NPs. Except for Ivermectin, it binds at the DGBM site with lower binding free energy (-6.7 to -7.4 kcal/mol) (Tables S4-3).

Three proposed NPs, Artemisinin (Supplementary, Fig. S2B), Ivermectin (Supplementary, Figure S2C), and DEG-168 (Supplementary, Fig. S2D) showed clear superiority over Remdesivir in simultaneous inhibition of both predicted binding sites. Additionally, Ivermectin and DEG-168 exhibited more affinity than Artemisinin the predicted binding sites. Meanwhile, even though DEG-168 inhibited more of the predicted binding residues, Ivermectin had a unique advantage in controlling the predicted binding sites due to its higher affinity for them (Supplementary, Fig. S2C and D).

**Figure 6:**
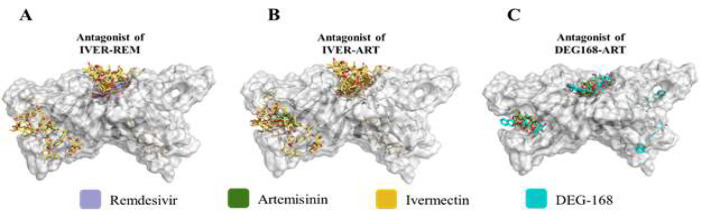
Demonstration of the antagonists between NPs due to the spatial interface of binding poses and greater binding affinity of Ivermectin and DEG-168. A) Lack of synergism between Remdesivir and Ivermectin. B) Lack of synergism between Artemisinin-Ivermectin. C) Lack of synergism between DEG168-artemisinin. The structure of the SARS-CoV-2 ORF8 homodimer is shown as a cartoon and transparent surface diagrams in gray color, the structures of Remdesivir, Artemisinin, Ivermectin, and DEG-168 are shown in stick diagram. REM, ART, IVER, and DEG stand for Remdesivir, Artemisinin, Ivermectin, and DEG-168, respectively.

**Figure 7:**
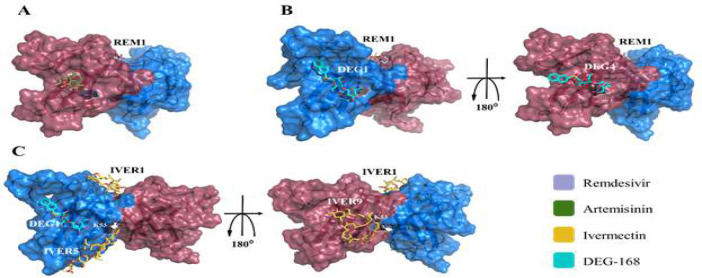
Co-functionality patterns of NPs. A) Remdesivir binding poses 1 in cooperation with Artemisinin binding poses 2. B) Remdesivir binding poses 1 in cooperation with DEG-168 binding poses 1 and 4. For more details. C) Ivermectin binding poses 1, 5, and 9 in cooperation with the DEG-168 binding pose 1. The dimeric structure of SARS-CoV-2 ORF8 is presented with a transparent surface, and its chains are shown in cartoon presentation and colored marine and raspberry for chains A and B, respectively. The suggested NPs Remdesivir, Artemisinin, Ivermectin, and DEG-168 have been displayed on sticks. Also, they have been colored in very peri, smudge, cyan, and gold, respectively. REM, ART, IVER, and DEG refer to Remdesivir, Artemisinin, Ivermectin, and DEG-168, respectively, and the numbers after them indicate NPs' binding pose number. Key residue K53 has been presented on the stick and labeled.

The use of combination therapy offers significant advantages due to the complementary coverage provided by each drug for the pharmacodynamic and pharmacokinetic limitations of the other. This is especially beneficial for targets like ORF8, which have wide-ranging intracellular and extracellular pathogenic functions. Combining solo-functionality patterns of NPs provided an insight into the co-functionality of these macromolecules (Figures 6 and 9). Ivermectin is antagonistic to Remdesivir and Artemisinin due to its higher affinity and the spatial overlap of its binding modes with those of Remdesivir and Artemisinin (Fig. 6 A to C). Consequently, three potent couple co-functionality patterns Including Remdesivir-Artemisinin, Remdesivir-DEG-168, and Ivermectin-DEG-168 were analyzed to overcome ORF8 (Figure 7 and supplementary, Table S3).

Both Artemisinin and DEG-168, in combination with Remdesivir, cover the defect of this COVID-19-approved NP-inspired drug in inhibiting the Gal1-like binding site. Additionally, the combination of DEG-168 with Ivermectin can be practical due to the inhibition of DGBM with a higher affinity than any other NPs (Figure 7), and the inhibition of more binding residues of the Gal1-like binding site (supplementary, Table S3). Also, the abundance of ORF8 residues has been controlled in this dual-combination therapy.

Table S3 in supplementary provides a general comparison of the potency of Remdesivir, Artemisinin, Ivermectin, and DEG-168 solo-functionalities and co-functionalities in terms of the ability to limit the most predicted binding residues.

The restrained residues, including predicted binding residues in both solo and combined functionalities of NPs, are listed and categorized in Table S3 (supplementary). E92 is prominently important, confirmed by its involvement in the solo functionality of all NPs, making it the most crucial residue in the DGBM. Additionally, interactions involving Q91, F120, and especially A51 of the DGBM are significant, as shown in Figure 5.

Notably, the DGBM binding residues G50, L95, G96, and S97 do not participate in the solo functionality of any of the four NPs. For the predicted Gal1-like binding site, R101 plays a crucial role in the interactions. Additionally, Y111 may be important for Gal1-like functions, which were previously underestimated in the statistical analysis of the participation rate of ORF8 interacting residues.

## DISCUSSION

Structural drug design studies have focused on the spike protein of the virus, while accessory proteins, especially SARS-CoV-2 ORF8, often play essential roles in immune modulation, host environment optimization, and viral pathogenicity [4, 9, 13]. Studies have shown that ORF8 in SARS-CoV-2 plays several important roles in disrupting the cellular machinery, regulating and evading the immune system, and even causing direct damage to host organ tissue [9, 14-19]. Despite the importance of ORF8, a limited number of inhibitors have been proposed for this protein. Few binding sites for ORF8 have been suggested or reported in some studies, but there are many other structural aspects for this multi-function protein [4, 31, 32].

Aiming to find potential binding sites for ORF8, analysis of the ORF8 structure revealed that a deep polar groove, dominated by negative charge, between two monomers (DGBM) has the hallmarks of a potential binding site (Fig. 2). Furthermore, I-TASSER server predictions also suggested another binding site (Gal1-like) on the surface of protein monomers (Fig. 3 and supplementary, Table S2). Moreover, checking the conservation level of the residues of these binding sites confirmed their prediction (Table 1). Additionally, it was predicted that the chemical and structural characteristics of E92 residue in DGBM alongside R101 and Y111 residues in Gal1-like play a key role in the interaction mechanism of these binding sites and effectively interact with the proposed inhibitors.

Investigating the conservation of the predicted binding site residues is crucial. As shown in Table 1, nearly all binding site residues are conserved at over 97% across all sequences, with the exception of F120, which is conserved at 85%. This indicates that the predicted binding sites are promising. 

Additionally, the rarity of these mutations’ co-occurrence supports the well-conserved structural fold in the mutated ORF8 variants [10, 99]. This study demonstrates that the predicted binding sites are highly conserved, suggesting that future research targeting ORF8 should focus on these sites. Subsequently, the results of four proposed natural products and ORF8 blind docking showed that in most of the generated complexes (32 out of 36 cases for all natural products), the ligand is docked on the binding sites of DGBM and Gal1-like, which confirms the accuracy of the prediction (Fig. 4). These complexes also show significantly lower binding free energies (-6.7 to -8.4 kcal/mol, Fig. 4 B).

In line with the fact that the DGBM binding site is inhibited frequently by ligands is highly frequent (26 out of 32 binding modes), DGBM is connected with the covalent interface. Inhibition of this interface can reduce the stability of high-order assemblies of this protein. This instability has been shown to be involved in the interaction of this protein with MHC-1 and the regulation of the immune system. 

The results of investigating solo-functionality patterns of NPs showed that both Ivermect and DEG-168 inhibited A51 and F120 simultaneously, and Remdesivir simultaneously inhibited all three residues of A51, R115, and F120. Previously, it was shown that the interaction of R115 of each chain with D119 of another chain stabilizes the core of the covalent interface. Additionally, the interaction of A51 of one chain with F120 of another chain also contributes to the dimer stability [31]. The interaction of these key residues with Remdesivir, Ivermectin, and DEG-168 (Figs. 8A, 8C, and 8D) probably interferes with their natural interaction network, leading to disrupting the spatial conformation of the covalent interface. This interference impacts the ORF8-MHC-I interaction negatively, leading to a reduction in immune evasion caused by ORF8 [32, 97].

Three proposed natural product inhibitors, Artemisinin, Ivermectin, and DEG-168, showed higher binding capacity and structural flexibility than Remdesivir. The blind docking results indicate that these three natural products bind to Gal1-like binding sites and other important residues in addition to DGBM, while Remdesivir only binds to DGBM. 

Moreover, Artemisinin, Ivermectin, and DEG-168 each individually demonstrated the ability to inhibit both predicted binding sites, showing a significantly low binding free energy range of (-6.7 to -8.4 kcal/mol).

The inhibition of all the predicted binding sites in the dimeric ORF8 by Ivermectin, which has a higher binding affinity score than other proposed natural products, supports the idea of the high efficiency of macrocyclic compounds in inhibiting ORF8.

The lactoside inhibitor DEG-168 showed the ability to inhibit V62 at its highest affinity score. The V62L mutation in ORF8 has been observed in several studies to be associated with mild disease and attenuated inflammatory responses [100]. Therefore, inhibiting the V62 mutation with the natural product inhibitor DEG-168 could be a practical treatment for controlling SARS-CoV-2.

In summary, the unique structure of multi-function ORF8 provides several binding sites for macromolecules. Two important functional binding epitopes, DGBM and Gal-like site, were identified and validated in this study using various approaches. Additionally, three natural products—Artemisinin, Ivermectin, and DEG-168—were proposed alongside the FDA-approved Remdesivir to inhibit and interrupt the functions of these binding sites. In addition to the advantages of natural products compared to synthetic chemicals, they can target ORF8 efficiency. Finally, key functional interacting residues were identified in the ORF8 structure.
