# Supplementary material for: Non-spike protein inhibition of SARS-CoV-2 by natural products through the key mediator protein ORF8
Source: Mol Biol Res Commun. 2025;14(1):73–91. doi: 10.22099/mbrc.2024.50245.2001 (PMC11624610; doi:10.22099/mbrc.2024.50245.2001)
Supplement: Supplementary file 1 — Figure S1 [file mbrc-14-73-s001.pdf]

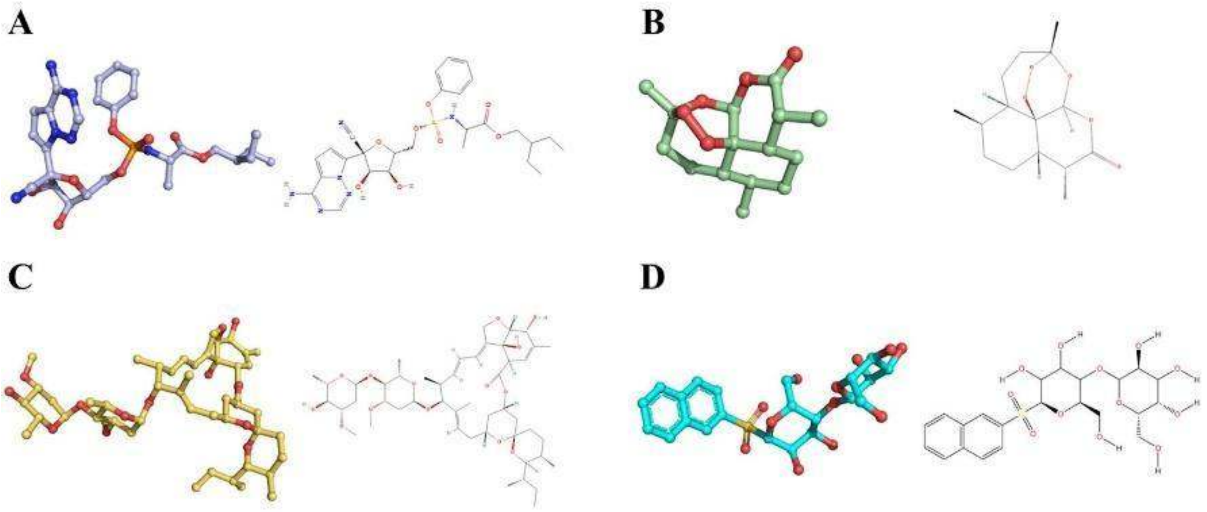

Figure S1: 2D and 3D presentation of four predicted potential NPs. A) Remdesivir, $C_{27}H_{35}N_6O_8P$, PubChem CID: 121304016. B) Artemisinin, $C_{12}H_{22}O_5$, PubChem CID: 68827. C) Ivermectin B1a, $C_{48}H_{74}O_{14}$, and PubChem CID: 21424, the 3D structure provided from PDB ID 5VDI. D) DEG-168, $C_{22}H_{28}O_{12}S$, the 2D and 3D structures are generated by ChemBioOffice software.

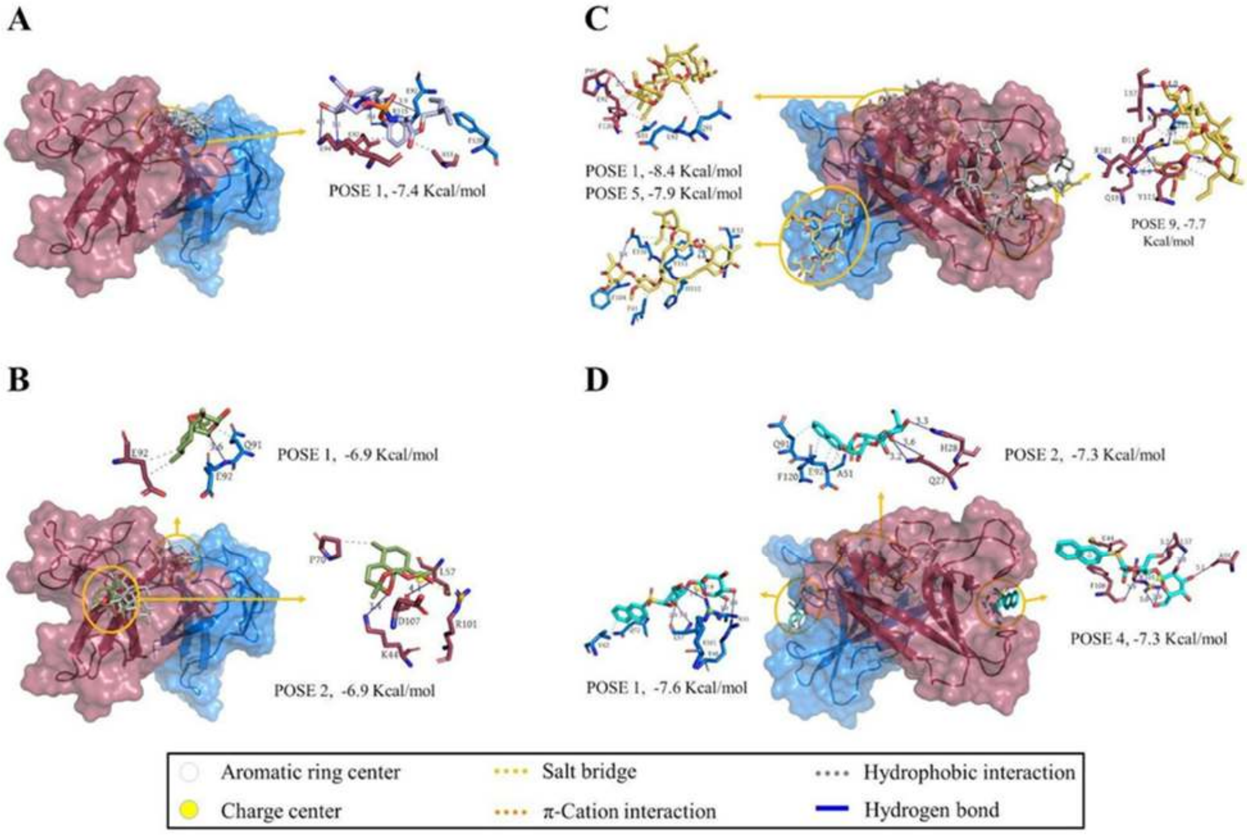

Figure S2: Solo-functionality patterns of NPs. A-D) Predicted Solo-functionalities of four suggested NPs between optimal binding modes. The dimeric structure of SARS-CoV-2 ORF8 is presented with a transparent surface and its chains are shown in cartoon presentation and colored marine and raspberry for chains A and B, respectively. The suggested NPs Remdesivir, Artemisinin, Ivermectin, and DEG-168 are displayed with very peri, smudge, cyan, and gold sticks, and the interactor residues are colored like the chain color code to which they belong and also are labeled. The color codes of interactions are to be declared below in a black box
