# Supplementary material for: Non-spike protein inhibition of SARS-CoV-2 by natural products through the key mediator protein ORF8
Source: Mol Biol Res Commun. 2025;14(1):73–91. doi: 10.22099/mbrc.2024.50245.2001 (PMC11624610; doi:10.22099/mbrc.2024.50245.2001)
Supplement: Supplementary file 2 — Table S1 [file mbrc-14-73-s002.pdf]

Table S1: shows the calculated Accessible Surface Area (ASA) for the residues forming the deep groove between the two monomers (DGBM) of ORF8. This criterion was computed using the Accessible Surface Area and Accessibility Calculation for Protein (ver. 1.2) server. The letters i, o, and i-o correspond to the ASA positions of the respective residues inside, outside, and in-between these two states in DGBM.

AMINO ACID	CHAIN	Area(Å2)	Relative (0.0 - 1.0)	IN/OUT
**Q27**	A	51.395	0.275	i-o
	B	35.704	0.191	i-o
**H28**	A	113.382	0.567	i-o
	B	104.953	0.524	i-o
**G50**	A	38.077	0.426	i-o
	B	33.742	0.377	i-o
**A51**	A	7.49	0.062	i
	B	17.25	0.143	i
**Q91**	A	30.536	0.163	i-o
	B	39.66	0.212	i-o
**E92**	A	42.778	0.227	i
	B	46.285	0.246	i
**P93**	A	9.276	0.06	i
	B	13.627	0.088	i
**K94**	A	119.708	0.56	i-o
	B	92.338	0.432	i-o
**L95**	A	31.634	0.153	i-o
	B	30.612	0.148	i-o
**G96**	A	18.455	0.206	i
	B	6.772	0.076	i
**S97**	A	0.113	0.001	i
	**B**	**2.951**	**0.023**	i
**R115**	A	3.519	0.015	i
	**B**	**5.387**	**0.023**	i
**F120**	A	7.674	0.034	i
	**B**	**8.503**	**0.037**	i

Table S2: TOP RESULTS OF I-TASSER SERVER PREDICTIONS FOR ORF8 BINDING SITES RESIDUES AND ITS NATIVE LIGANDS. These two were selected among five predicted templates in the I-TASSER server. The I-TASSER has achieved it by using COACH and CO-FACTOR servers. C-score is refer to the confidence score of the prediction and its ranges [0-1], where a higher score indicates a more reliable prediction. S.S refers to the secondary structure in which binding residues are located there on the SARS-CoV-2 ORF8 3D crystallography structure (FIG 1).

NO	TEMPLATE PDB ID/CHAIN	INTRODUCTION	LIGAND	C-score	BINDING SITE/S.S					
1	4y1x/A 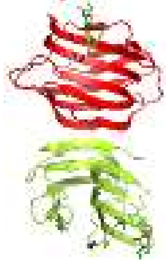	Complex of human Galectin-1 (Gal-1) and Galbeta1-4(6OSO3)GlcNAc	6S2	0.07	R48/β3	–	R101/ β7	Y111/ β8	D113/ β8	R115/ β8
2	1y0l/H 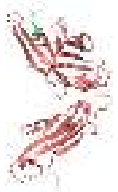	Catalytic elimination antibody 34E4 in complex with hapten	HAN	0.07	–	E59/β4	R101/ β7	Y111/ β8	–	–

Table S3. The residues inhibited in NPs predicted solo and co-functionalities. The numbers after the NPs' name refer to optimal binding poses that each NP inhibits predicted binding sites. The letters A and B in cells refer to the ORF8 chain

INHIBITED RESIDUES		PREDICTED BINDING SITE RESIDUES																		OTHER RESIDUES																	
		DGBM													Gal1-like																					
		Q27	H28	G50	A51	Q91	E92	P93	K94	L95	G96	S97	F120	R115†	R48	E59	R101	Y111	D113	Q18	F41	K44	K53	A55	L57	V62	S67	P70	Q72	F104	D107	F108	Y109	E110	H112	I121
NPs Solo-Functionality	Remdesivir 1				● B		● A B		● B				● A	● A																						
	Artemisinin 1,2					● A	● A B										● B					● B			● B			● B			● B					
	Ivermectin 1,5,9				● A	● A	● A B	● B					● B				● B	● A B	● B	● B	● A		● A B		● B		● B			● A	● B		● B	● A	● A	● A
	DEG-168 1,2,4	● B	● B		● A	● A	● A						● A		● A		● A B	● A B				● B		● A B	● A B	● A			● A			● B				
NPs Co-Functionality	Remdesivir 1-Artemisinin 2				● B		● A B		● B				● A	● A			● B					● B			● B			● B			● B					
	Remdesivir 1-DEG168 1, 4				● B		● A B		● B				● A	● A	● A		● A B	● A B				● B		● A B	● A B	● A			● A			● B				
	Ivermectin 1,5,9-DEG168 1				● A	● A	● A B	● B					● B		● A		● A B	● A B	● B	● B	● A		● A B		● B	● A	● B		● A	● A	● B		● B	● A	● A	● A

† Residue R115 is predicted for both binding sites, but in the minimized structure it was accessible and exposed only at the DGBM binding site, so it was considered for this binding site.

Table S4: PREDICTED BINDING SITE RESIDUES AND THEIR CONSERVATION. Conservation has been calculated in Jalview software (101) by the AMAS method of multiple sequence alignment analysis (102) for 22947 aligned SARS-CoV-2 ORF8 sequences. AMSA scores the level of conservation of amino acid residues from 0-11 (from low to high). A score of 11 indicates grouping with default amino acid property, and a score of 10 indicates mutations that all default amino acid properties are conserved. SARS-CoV-2 ORF8 sequences had been aligned by MEGA7 (99) software using the MUSCLE algorithm (100)

NO	BINDING SITE SPECIFY METHOD		BINDING SITE RESIDUE NAME & NUMBER	MUTATION/MUT. FREQUENTLY%	CONSERVATION (0-11)	~CONSENSUS%
	**I-TASSER Prediction**	**Structural Analyses**				
1		●	**Q27**	**R/0.004, K/0.004, Gap**	**10**	**99**
2		●	**H28**	**L/0.004, Y/0.004, Gap**	**10**	**99**
3		●	**G50**	**Gap**	**11**	**99**
4		●	**A51**	**S/1.32, V/0.109, F/0.013,T/0.013, Gap**	**10**	**98**
5		●	**Q91**	**K/0.009, I/0.004, L/0.004, P/0.004, R/0.004, Gap**	**10**	**99**
6		●	**E92**	**K/1.286, S/0.004, Gap**	**10**	**98**
7		●	**P93**	**S/2.244, L/0.01, Gap**	**10**	**97**
8		●	**K94**	**E/0.009, R/0.004, Gap**	**10**	**99**
9		●	**L95**	**F/0.253, M/0.004, S/0.004, V/0.004, Gap**	**10**	**99**
10		●	**G96**	**D/0.004, Gap**	**10**	**99**
11		●	**S97**	**I/0.009, G/0.004, Gap**	**10**	**99**
12		●	**F120**	**L/0.689, K/0.022, V/0.017, I/0.004, Gap/14.2**	**2**	**85**
13	●	●	**R115**	**C/0.065, L/0.026, P/0.017, H/0.013, Gap**	**10**	**99**
14	●		**R48**	**I/0.004, Gap**	**10**	**99**
15	●		**E59**	**V/0.013, G/0.004, K/0.004, Gap**	**10**	**99**
16	●		**R101**	**L/0.022, Gap**	**10**	**99**
17	●		**Y111**	**C/0.004, Gap**	**10**	**99**
18	●		**D113**	**Y/0.004, Gap**	**10**	**99**

previously. R115 was predicted by both methods, but in our minimized ORF8 model, it was only exposed in the DGBM binding site.

Table S5: MOLECULAR DOCKING RESULTS FOR ORF8-REMDESIVIR COMPLEXES. The results are sorted from highest to lowest based on the binding affinity score ($Kcal.mol^{-1}$) from AutoDock Vina's output. Interacting residues of the predicted DGBM and Gal1-like binding sites are highlighted in green color and solid blue triangles, respectively. The letters A and B after the residue number refer to the chain where the residue is located. The R.46-83 is a structurally unique region of ORF8 located between residues 46-83.

BINDING POSE	SCORE $Kcal.mol^{-1}$	Lower Bond RMSD	INVOLVED RESIDUES IN H.bond	DISTANCE D-Å	INVOLVED RESIDUES IN HYDROPHOBIC INTERACTION	INVOLVED RESIDUES IN SALT BRIDGE INTERACTION	LOCATION ON ORF8
**1**	-7.4	0	92A<br>92B<br>94B<br>115A	3.94<br>3.09<br>4.06<br>3.44	51B<br>92A<br>92B<br>120A<br>120A	-	DGBM
**2**	-7.2	3.318	27A<br>93A<br>93B<br>96A	3.24<br>3.12<br>2.7<br>3.83	51A<br>91A<br>92A<br>94A<br>120B	-	DGBM
**3**	-7.0	2.706	27B<br>92A<br>92B<br>93A<br>115A<br>115A	4.02<br>3<br>3.92<br>3.81<br>3.25<br>4	51B<br>91A<br>92B<br>94A	-	DGBM
**4**	-6.9	2.521	27B<br>50A<br>93B<br>94B	3.74<br>3.37<br>2.97<br>3.66	51A<br>51B<br>92A	-	DGBM
**5**	-6.9	3.259	92B<br>115A<br>115A	3.65<br>3.84<br>3.32	51B<br>91B<br>92A<br>92A<br>92B<br>120B	-	DGBM
**6**	-6.8	1.936	92A<br>92B<br>94B<br>115A	3.78<br>2.92<br>3.98<br>3.46	92A<br>92A<br>120A	-	DGBM
**7**	-6.8	2.235	91B<br>94B	3.81<br>3.24	51A<br>51B<br>91A<br>92A<br>92B<br>92B<br>120A<br>120A	-	DGBM

					120B		
**8**	-6.7	3.192	27A<br>92A<br>92A<br>96B<br>115A	3.43<br>3.74<br>3.44<br>3.11<br>3.46	51B<br>92B<br>94A<br>120A	28A	DGBM
**9**	-6.7	2.949	27A<br>91A<br>92A<br>96B	3.01<br>3.92<br>3.07<br>2.8	51B<br>91B<br>92B	-	DGBM

Table S6: MOLECULAR DOCKING RESULTS FOR ORF8-ARTEMISININ COMPLEXES. The results are sorted from highest to lowest based on the binding affinity score (Kcal.mol-1) from AutoDock Vina's output. Interacting residues of the predicted DGBM and Gal1-like binding sites are highlighted in green color and solid blue triangles, respectively. The letters A and B after the residue number refer to the chain where the residue is located. The R.46-83 is a structurally unique region of ORF8 located between residues 46-83.

BINDING POSE	SCORE $Kcal.mol^{-1}$	Lower Bond RMSD	INVOLVED RESIDUES IN H.bond	DISTANCE D-Å	INVOLVED RESIDUES IN HYDROPHOBIC INTERACTION	INVOLVED RESIDUES IN SALT BRIDGE INTERACTION	LOCATION ON ORF8
**1**	-6.9	0	92A	3.63	91A<br>92B<br>92B	-	DGBM
**2**	-6.9	21.372	44B<br>107B	3.45<br>4.08	57B<br>70B	101B ▲	R.46-83
**3**	-6.8	1.577	92B	3.9	51B<br>91A<br>92A<br>92B	-	DGBM
**4**	-6.8	19.066	-	-	56B<br>57B<br>57B<br>57B<br>108B	101B ▲<br>101B ▲	R.46-83
**5**	-6.7	6.176	-	-	91B<br>120B	-	DGBM
**6**	-6.7	20.846	-	-	57B<br>57B	44B	R.46-83
**7**	-6.7	2.874	93A	2.85	51B<br>91A	-	DGBM
**8**	-6.6	2.524	93B	2.85	51B<br>91A<br>92A<br>120A	-	DGBM
**9**	-6.5	5.237	92B	3.23	92A<br>92B<br>94B<br>120B	115A	DGBM

Table S7: MOLECULAR DOCKING RESULTS FOR ORF8-IVERMECTINE COMPLEXES. The results are sorted from highest to lowest based on the binding affinity score (Kcal.mol-1) from AutoDock Vina's output. Interacting residues of the predicted DGBM and Gal1-like binding sites are highlighted in green color and solid blue triangles, respectively. The letters A and B after the residue number refer to the chain where the residue is located. The R.46-83 is a structurally unique region of ORF8 located between residues 46-83.

BINDING POSE	SCORE Kcal.mol^{-1}	Lower Bond RMSD	INVOLVED RESIDUES IN H.bond	DISTANCE D-Å	INVOLVED RESIDUES IN HYDROPHOBIC INTERACTION	INVOLVED RESIDUES IN SALT BRIDGE INTERACTION	LOCATION ON ORF8
**1**	-8.4	0	93B	2.74	51A<br>91A<br>92A<br>92B<br>92B<br>120B<br>120B	-	DGBM
**2**	-8.3	4.3	27A<br>28A	3.01<br>3.84	51A<br>51B<br>91A<br>92A	94B	DGBM
**3**	-8.2	25.582	67B<br>73B	3.85<br>2.81	57B<br>73B<br>109B<br>109B	44B	R.46-83
**4**	-8.0	2.904	92B<br>96A	2.92<br>3.78	51A<br>51B<br>76B<br>91A<br>91B<br>92A<br>92A<br>92B<br>94A<br>94B<br>120A<br>120A	-	DGBM
**5**	-7.9	22.379	110A<br>111A ▲<br>111A ▲	3.44<br>3.96<br>3.96	41A<br>41A<br>53A<br>104A<br>110A<br>110A<br>111A ▲<br>111A ▲<br>112A	112A	R.46-83
**6**	-7.8	3.045	92B<br>94B	2.86<br>3.9	51A<br>51B<br>76B<br>92B<br>92B<br>94A<br>120A<br>120B	-	DGBM
**7**	-7.7	2.729	27A	2.94	51A<br>91B	94B	DGBM
**8**	-7.7	26.831	43B<br>61B<br>64B	3.04<br>3.73<br>4.06	45B<br>86B<br>108B	-	R.46-83
**9**	-7.7	23.858	57B<br>101B ▲<br>111B ▲<br>113B ▲<br>113B ▲	4.03<br>3.45<br>3.77<br>2.9<br>3.95	18B<br>53B<br>53B<br>111B ▲<br>121A	101B ▲	R.46-83

Table S8: MOLECULAR DOCKING RESULTS FOR ORF8-DEG-168 COMPLEXES. The results are sorted from highest to lowest based on the binding affinity score (Kcal.mol-1) from AutoDock Vina's output. Interacting residues of the predicted DGBM and Gal1-like binding sites are highlighted in green color and solid blue triangles, respectively. The letters A and B after the residue number refer to the chain where the residue is located. The R.46-83 is a structurally unique region of ORF8 located between residues 46

BINDING POSE	SCORE Kcal.mol^{-1}	Lower Bond RMSD	INVOLVED RESIDUES IN H.bond	DISTANCE D-Å	INVOLVED RESIDUES IN HYDROPHOBIC INTERACTION	INVOLVED RESIDUES IN SALT BRIDGE INTERACTION	INVOLVED RESIDUES π-CATION INTERACTIONS	LOCATION ON ORF8
**1**	-7.6	0	48A ▲<br>55A<br>57A<br>57A<br>101A ▲<br>101A ▲	3.78<br>3.02<br>3.3<br>2.86<br>2.92<br>3.3	62A<br>72A	101A ▲	-	R.47-83
**2**	-7.3	19.258	27B<br>27B<br>28B	3.18<br>3.65<br>3.31	51B<br>91A<br>92A<br>92A<br>120A	-	-	DGBM
**3**	-7.3	23.747	27A<br>50B<br>96B	3.52<br>3.69<br>3.46	91B<br>92B<br>92B<br>120B	-	-	DGBM
**4**	-7-2	40.495	55B<br>57B<br>57B<br>101B ▲<br>101B ▲<br>108B	3.11<br>3.24<br>2.85<br>3<br>3.94<br>3.89	108B	101B ▲	44B	R.47-83
**5**	-7.2	18.651	27B<br>27B<br>28B<br>50A<br>93A<br>95A	3.08<br>3.68<br>3.01<br>4.08<br>3.72<br>4.09	91A<br>120A	-	-	DGBM
**6**	-7.2	16.323	43A<br>45A<br>45A<br>45A<br>60A<br>83A<br>83A<br>86A	2.99<br>2.7<br>3.71<br>3.09<br>3.28<br>3.1<br>2.83<br>3.06	32A<br>85A	-	-	R.47-83
**7**	-7.0	18.103	28B<br>50A	3.68<br>3.73	51B<br>92B<br>92B	-	-	DGBM
**8**	-7.0	22.834	50A<br>92A<br>92B<br>95A	3.39<br>3.77<br>3.18<br>3.95	-	-	-	DGBM
**9**	-7.0	18.801	27B<br>28B<br>92B<br>93A<br>95A<br>96A<br>96A	3.45<br>3.94<br>3.43<br>3.82<br>3.69<br>3.37<br>2.79	51B<br>91A<br>92A<br>92A	-	-	DGBM
